# Natural Si/N Co-Doped Porous Biomass Carbon Micron-Tubes as High-Performance Anode Materials for Lithium-Ion Batteries

**DOI:** 10.3390/ma19142951

**Published:** 2026-07-09

**Authors:** Ziqing Xu, Kai Cao, Zhifeng Wang

**Affiliations:** “The Belt and Road Initiative” Advanced Materials International Joint Research Center of Hebei Province, School of Materials Science and Engineering, Hebei University of Technology, Tianjin 300401, China; 202331802063@stu.hebut.edu.cn (Z.X.); caokairesearch@163.com (K.C.)

**Keywords:** biomass carbon, lithium-ion battery, anode, Si/N co-doping

## Abstract

The development of carbon-based anode materials for high-performance lithium-ion batteries has been limited by their low theoretical capacity density, low conductivity, and high manufacturing costs. Herein, natural Si/N co-doped biomass carbon micron-tubes, derived from reed catkins, were synthesized. The as-prepared RC-Si/N anode exhibits a good discharge capacity of 761.3 mAh g^−1^ at 100 mA g^−1^ after 200 cycles. Moreover, it exhibits outstanding cycling stability, retaining discharge capacities of 517.7 mAh g^−1^ at 1 A g^−1^ after 1000 cycles. The excellent electrochemical performance is attributed to the trace Si originating from the biomass precursor, which provides high specific capacity, while N doping introduces structural defects and improves electronic conductivity. Coupled with its unique micrometer-scale tubular morphology, the material facilitates efficient lithium-ion transport and storage. Further DFT calculations corroborate enhanced Li^+^ adsorption ability, sustained structural integrity over prolonged cycling, and promoted reaction kinetics. These findings underscore the potential of natural Si/N co-doped biomass-derived carbon as an advanced lithium-ion battery anode material.

## 1. Introduction

Given the pressing issues of environmental pollution and the over-reliance on fossil fuels [1,2,3,4,5], sustainable energy technologies have witnessed remarkable progress [6,7,8,9]. In this context, lithium-ion batteries (LIBs) have become the focus of extensive research [10,11]. As a crucial constituent of LIBs, anode materials substantially govern the key performance metrics of the batteries [12,13]. Although graphite has served as the conventional anode material owing to its excellent reversibility [14,15], its constrained theoretical capacity (372 mAh g^−1^) and inherent electronic conductivity limitations have stimulated considerable efforts toward developing advanced carbon-based alternatives. Various carbon-related materials, including graphene, carbon nanospheres, and carbon nanotubes [16,17,18,19,20,21], have been developed and have demonstrated excellent lithium storage performance. However, the manufacturing cost of these carbon materials is extremely high, which significantly hinders their progress toward market introduction. Developing novel carbon materials that are both low cost and high-performance remains a significant challenge.

In recent years, carbon materials derived from biomass sources have aroused widespread research interest owing to their cost-effectiveness, renewability, environmental sustainability, and natural abundance. Various naturally existing carbon precursors [22,23,24,25,26,27,28,29,30] have been applied in various energy storage systems. Li et al. fabricated porous spherical carbon materials from lotus root as a LIB anode material, achieving a capacity of 187.2 mAh g^−1^ at 10 A g^−1^ and demonstrating outstanding rate performance [31]. Qin et al. fabricated a layered granular carbon material from Auricularia fungus chaff as a sodium-ion anode, delivering a specific capacity of 450 mAh g^−1^ and a first Coulombic efficiency of 81% at 0.1 C, along with stable cycling performance [32]. Abhishek Dharmesh et al. developed a porous, block-shaped carbon material from tamarind peel as a lithium-sulfur battery cathode, which presents 1265 mAh g^−1^ at 0.1 C and maintains high Coulombic efficiencies around 98% over 300 cycles [33]. Yang et al. fabricated a hierarchical porous material from sugarcane bagasse and hydroxyapatite, which demonstrated a specific capacitance of 397.9 F g^−1^ at 0.5 A g^−1^ when applied in supercapacitors, showing excellent specific capacitance [34]. Through extensive research, the development of biomass-derived carbon has significantly reduced the manufacturing cost of electrode materials. Nevertheless, the practical deployment of biomass-related carbon in LIBs remains hampered by certain limitations, such as low conductivity and inadequate specific capacity.

To address these limitations, considerable research efforts have been directed toward structural engineering and heteroatom doping, as well as the formation of composites with high-capacity anode materials. Liu et al. extracted cellulose as a carbon source to synthesize block-structured carbon materials. They further employed structural engineering strategies to assemble highly disordered carbon materials via hydrothermal processing in a zinc chloride solution. The product exhibited a capacity of 425 mAh g^−1^ at 0.1 A g^−1^ and demonstrated outstanding stability in lithium-ion batteries [35]. Han et al. fabricated porous carbon materials using cellulose as a carbon source through a heteroatom doping strategy. Phosphorus dopants were introduced into the carbon materials via freeze-drying. The product uncovered a high lithium storage capacity of 1848 mAh g^−1^ at 0.1 A g^−1^ [36]. Sang synthesized block-structured carbon materials using stone flower algae as a carbon precursor. Subsequently, a freeze-drying method was employed to form a composite material by combining the carbon material with higher-capacity Si@NiO/Ni. This composite displayed a capacity of 1245.1 mAh g^−1^ at 1 A g^−1^ [37]. Although the aforementioned strategies have improved the electrochemical performance of carbon materials to some extent, they introduce additional processing steps that significantly diminish the low-cost manufacturing advantage of biomass-derived carbon. Therefore, it remains highly challenging to implement strategies such as structural engineering and heteroatom doping to enhance lithium storage performance without increasing manufacturing costs or introducing extra processing steps.

In this study, biomass carbon micron-tubes inherently doped with natural silicon and nitrogen elements were prepared directly through carbonization of reed catkins without additional treatment. The as-prepared RC-Si/N anode, with micron-tube structure and Si/N dual doping, provides unique direct conductive pathways that enable faster electron transport and reaction kinetics, and enhanced reversible capacity contributed by Si elements. Thus, the RC-Si/N anode exhibits outstanding capacity and cycling stability, delivering a specific discharge capacity of 761.3 mAh g^−1^ after cycling at 0.1 A g^−1^ for 200 cycles, and even retaining 517.7 mAh g^−1^ after 1000 cycles at 1 A g^−1^. This work highlights the significant importance of developing biomass-derived carbon materials and serves as a reference for creating more low-cost carbon materials and advancing their application in energy storage.

## 2. Materials and Methods

**Figure 1:**
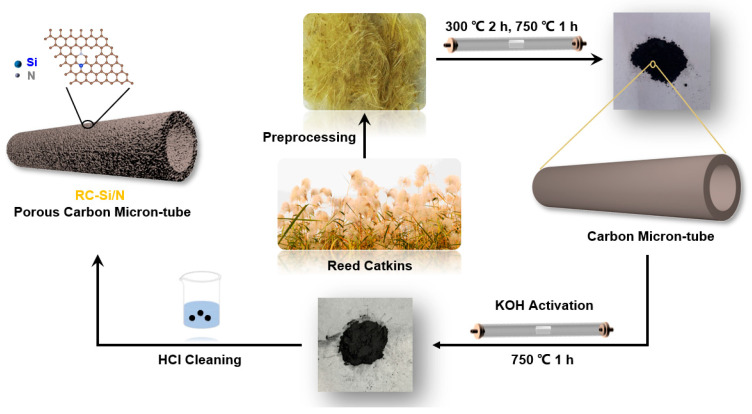
The preparation process of RC-Si/N.

The synthesis of RC-Si/N commenced with the purification of harvested reed catkins, which were ultrasonically cleaned in deionized water and subsequently dried at 80 °C for 12 h. The resulting powders were then subjected to a two-step pyrolysis process under an argon atmosphere. They were first heated to 300 °C at a heating rate of 3 °C min^−1^ and kept for 2 h, then further elevated to 750 °C and held for 1 h, followed by natural furnace cooling. The resulting carbonized intermediate was thoroughly and homogeneously mixed with KOH at a mass ratio of 1:4. The mixture underwent a second heating at 750 °C for 1 h under argon with the same heating rate, then cooled to ambient temperature. The final materials were cleaned with 1 M HCl for 12 h to remove KOH and impurities, then rinsed repeatedly with distilled water until a neutral pH was achieved, and finally dried at 100 °C for 12 h to obtain the RC-Si/N material, as schematically illustrated in Figure 1. To elucidate the role of naturally occurring silicon, a contrast sample designated as RC-N was prepared by etching RC-Si/N with 1 M HF for 12 h to remove Si elements. Furthermore, commercial graphite (Aladdin, Shanghai, China, CAS: 1333–86–4) was also used as a contrast material. Other experimental details, including material characterization, electrochemical measurements, and calculation method, can be found in Supporting S1 (Experimental Details).

## 3. Results and Discussion

### 3.1. Material Characterizations

**Figure 2:**
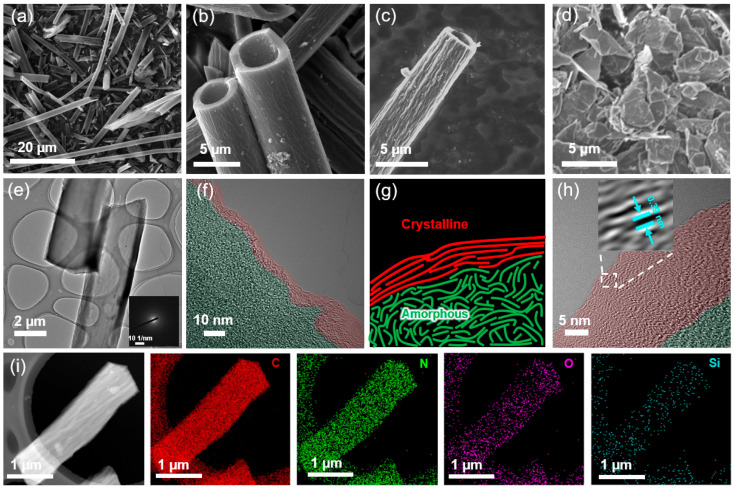
SEM images of (**a**,**b**) RC-Si/N, (**c**) RC-N and (**d**) Graphite; (**e**) TEM and SAED images of RC-Si/N; (**f**,**h**) HRTEM images of RC-Si/N; (**g**) The schematic diagram showing the surface structure of RC-Si/N; (**i**) EDS mapping of RC-Si/N.

The morphological and structural characteristics of the samples were investigated by SEM and TEM, as shown in Figure 2. SEM images of RC-Si/N reveal the presence of hollow tubular structures with outer diameters of approximately 5 µm (wall thickness around 1 μm), displaying a slightly textured surface in Figure 2a,b. In contrast, the HF-etched RC-N sample exhibits a roughened morphology with significantly enhanced microporosity (Figure 2c and Figure S1a,b). Energy-dispersive X-ray spectroscopy of RC-N validates the effective removal of silicon from RC-Si/N, with the remaining elements being C, N, and O (Figure S1c–f). Commercial graphite shows characteristic dense and irregular particles with negligible porosity in Figure 2d. Figure 2e shows the TEM image of the RC-Si/N anode, further confirming its hollow tubular structure. This structure is crucial for accommodating volume changes during long-term cycling while maintaining structural integrity. Additionally, the embedded diffraction rings in Figure 2e further confirm its amorphous structure. Figure 2f presents the HRTEM image of the RC-Si/N anode, revealing a predominantly disordered carbon structure with graphite-like features in certain regions. The schematic (Figure 2g) illustrates a thin graphitic-like surface layer coating on the carbon micron-tubes, while the bulk interior maintains an amorphous structure. Figure 2h shows a further magnified HRTEM image of the RC-Si/N anode, revealing graphite-like regions with an interlayer spacing of 0.39 nm. EDS mapping (Figure 2i) demonstrates the homogeneous dispersion of C, N, O, and Si across the RC-Si/N architecture, with a visually evident higher silicon content compared with the RC-N material.

**Figure 3:**
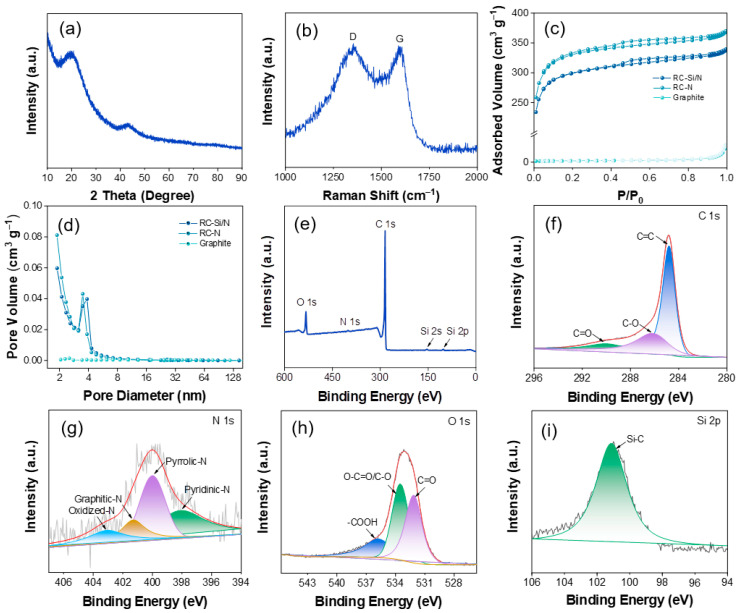
Microstructural characterization: (**a**) XRD pattern, (**b**) Raman pattern of RC-Si/N; (**c**) N_2_ adsorption/desorption profiles and (**d**) Pore size distributions of RC-Si/N, RC-N, and Graphite; (**e**) XPS spectra of RC-Si/N; High-resolution spectra of (**f**) C 1s, (**g**) N 1s, (**h**) O 1s, (**i**) Si 2p.

The crystalline structure of RC-Si/N was characterized by XRD (Figure 3a) and Raman spectroscopy (Figure 3b). The XRD pattern exhibits two broad diffuse peaks at about 20.01° and 43.22°, coinciding with the (002) and (100) planes of low-crystallinity/less ordered carbon, confirming its predominantly amorphous nature. As shown in Figure 2h, the interlayer spacing in the graphite-like region of the RC-Si/N anode is 0.39 nm, whereas that of graphite is 0.335 nm [38]. The widening of the lattice spacing is correlated with the KOH activation process, which involves the incorporation of potassium into the carbon layers, leading to lattice expansion [39]. The enlarged interlayer spacing facilitates reversible Li^+^ intercalation/deintercalation and enhances structural stability during cycling [40]. Raman spectroscopy further reveals the structural characteristics of RC-Si/N, with a distinct D band at ~1354 cm^−1^ arising from structural defects and disordered carbon, and a G band at ~1589 cm^−1^ linked to in-plane vibrations of sp^2^-hybridized carbon. The I_D_/I_G_ intensity ratio of 1.02 indicates a low degree of graphitization and a high concentration of defects, which is consistent with the HRTEM and SAED observations. These structural features contribute to improved electrochemical performance by providing abundant active sites.

The porous characteristics of the three materials were evaluated through N_2_ adsorption–desorption measurements, with the resulting isotherms and pore size distributions displayed in Figure 3c,d. The RC-Si/N and RC-N samples reflect typical Type IV isotherms with H4-type hysteresis curves, disclosing the typical mesoporous characteristics of the products. The obtained specific surface areas for RC-Si/N, RC-N, and graphite are 1157.1, 1273.9, and 7.3 m^2^ g^−1^, respectively. Correspondingly, the predominant pore sizes for these materials are centered at approximately 3.8 nm, 3.6 nm, and 30.6 nm. It should be noted that after HF etching of the RC-N material, the vast majority of Si is removed, thereby creating more micropores on the surface of the material [27]. Therefore, RC-N exhibits a slightly higher specific surface area than RC-Si/N, while commercial graphite shows a low-porosity feature.

The surface chemical analysis and bonding structures of the RC-Si/N material were analyzed by XPS. The full spectrum demonstrates the presence of C, N, O, and Si [41], with characteristic peaks observed at 285 eV (C 1s), 400 eV (N 1s), 533 eV (O 1s), 154 eV (Si 2s), and 102 eV (Si 2p) in Figure 3e. High-resolution spectra were deconvoluted to uncover the chemical bonding configurations. The C 1s spectrum was fitted into three components including C=C (284.8 eV), C-O (286.2 eV), and C=O (290.1 eV), as shown in Figure 3f. The N 1s spectrum reveals four nitrogen species relating to pyridinic N (398.1 eV), pyrrolic N (400.0 eV), graphitic N (401.3 eV), and oxidized N (403.1 eV) (Figure 3g) [42,43]. The presence of these nitrogen moieties, particularly graphitic N, significantly tunes the electronic properties of the carbon matrix by introducing defect sites and enhancing surface reactivity, which contributes to improved electrochemical performance. The O 1s spectrum was resolved into C=O (532.1 eV), C-O/O-C=O (533.6 eV), and -COOH (535.9 eV) in Figure 3h. While the Si 2p spectrum reveals a single peak at 101.1 eV, consistent with the Si-C bond, suggesting the successful incorporation of silicon into the carbon framework (Figure 3i) [44,45,46]. The presence of Si-C bonds contributes to enhanced electrocatalytic activity, promoting oxygen reduction reactions during cycling. This effect improves the electrochemical performance under high-rate conditions while maintaining excellent electrochemical stability. Furthermore, the synergistic interaction between silicon-carbon bonding and nitrogen doping induces remarkable pseudocapacitive behavior within the carbon matrix. The silicon content was quantitatively determined by ICP analysis, revealing values of 1.873–1.882 wt% for RC-Si/N and 0.018–0.019 wt% for RC-N across different batches, as listed in Table S1. This confirms the relative stability of the silicon content in the products.

### 3.2. Electrochemical Measurements

**Figure 4:**
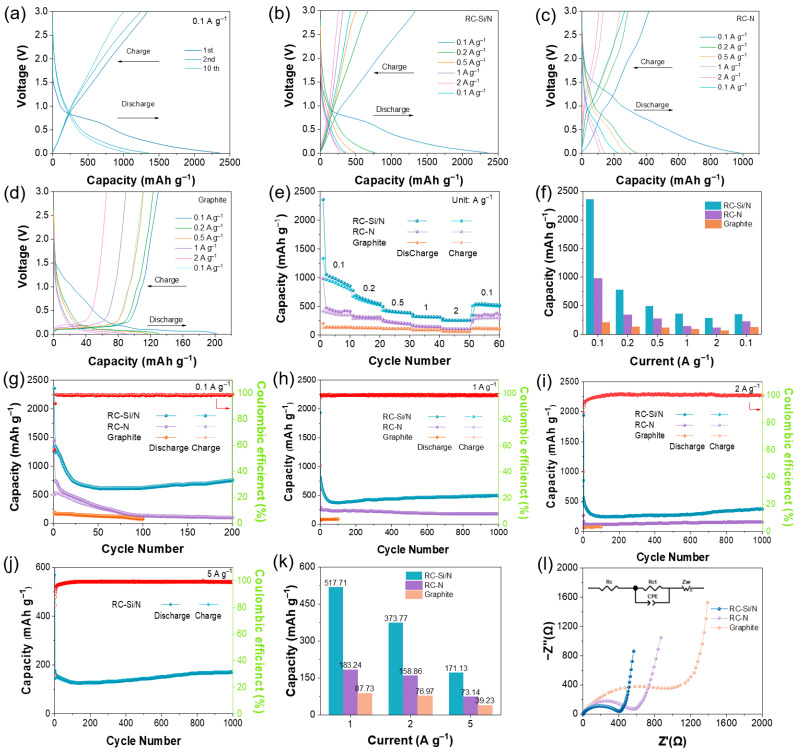
Electrochemical characterization: (**a**) Discharge/charge profile of the RC-Si/N for the 1st, 2nd, and 10th cycle at 0.1 A g^−1^; Galvanostatic discharge/charge profile of the (**b**) RC-Si/N, (**c**) RC-N and (**d**) Graphite anodes at various current densities; (**e**) Rate performance of three anodes; (**f**) Performance comparison of RC-Si/N, RC-N and Graphite anodes at 0.1, 0.2, 0.5, 1, 2, 0.1 A g^−1^; (**g**) Short-term cycling performance of three anodes at 0.1 A g^−1^; Long-term cycling performance of three anodes at (**h**) 1 A g^−1^, (**i**) 2 A g^−1^, and (**j**) 5 A g^−1^; (**k**) Comparison of the discharge capacities of RC-Si/N anodes and RC-N anodes after 1000 cycles and graphite anodes after 100 cycles measured at 1, 2, and 5 A g^−1^; (**l**) EIS curves of the three anodes.

As illustrated in Figure 4a, the galvanostatic charge–discharge curves of the RC-Si/N anode at 0.1 A g^−1^ for the 1st, 2nd, and 10th cycles are presented. The first cycle delivers discharge/charge capacities of 2357.7/1335.2 mAh g^−1^, corresponding to an initial Coulombic efficiency (ICE) of 56.63%. The electrode exhibits reversible capacities of 1350.2 mAh g^−1^ in the second cycle and 1087.5 mAh g^−1^ in the tenth cycle, demonstrating effective capacity retention and cycling stability. The low ICE of biomass-derived carbon anodes is primarily caused by the irreversible consumption of lithium ions during the formation of the solid electrolyte interphase (SEI) on abundant defective sites and high specific surface area, as well as the irreversible trapping of lithium within the closed micropores and disordered carbon structures. These parasitic reactions and lithium entrapment substantially reduce the number of recyclable lithium ions in the first cycle. Table S2 [24,47,48,49,50,51,52,53,54] compares the ICE values of the synthesized materials in this paper with those of other biomass-derived carbon materials reported in the literature. As can be seen from the table, without any improvement measures, the ICE of most materials is relatively low. To improve the low ICE of biomass-derived carbon anodes, different strategies such as pre-lithiation, adjustment of the voltage range, and electrolyte optimization [55,56,57] can be adopted. First, pre-lithiation is a direct and effective strategy, in which chemical or electrochemical lithium supplementation compensates for the lithium consumed during SEI formation, thereby raising the ICE above 80%. Voltage window optimization can also mitigate irreversible capacity by avoiding excessively low cut-off potentials (e.g., >0.01 V vs. Li/Li^+^) that promote electrolyte decomposition and active lithium trapping in micropores, while a slightly higher upper limit reduces side reactions. Electrolyte tuning, including the use of highly concentrated salts (e.g., LiFSI), fluorinated solvents, or functional additives such as FEC and VC, enables the formation of a thinner, more inorganic-rich, and stable SEI with lower ionic resistance and reduced lithium consumption. These approaches are often combined to synergistically enhance ICE without sacrificing capacity.

Figure 4b–d compare the initial charge–discharge profiles of RC-Si/N, RC-N, and graphite at 0.1 to 2.0 A g^−1^. Across all tested rates, RC-Si/N maintains the highest electrochemical performance among the three materials. The rate capabilities are further compared in Figure 4e, which summarizes the cycling performance over a rate range from 0.1 to 2 A g^−1^. After 10 cycles at each rate condition, the RC-Si/N electrode delivered reversible capacities of 854.5, 546.1, 389.4, 314.7, and 256.8 mAh g^−1^ at 0.1, 0.2, 0.5, 1, and 2 A g^−1^, respectively. Significantly, upon returning the rate to 0.1 A g^−1^, the capacity recovered to 522.8 mAh g^−1^ (Figure 4e), highlighting its exceptional rate capability and structural reversibility. By contrast, the HF-etched RC-N anode yielded lower specific capacities of 413.4, 317.2, 198.0, 140.9, and 61.3 mAh g^−1^ at the respective current densities, while a recovery value of 355.5 mAh g^−1^ was observed upon returning to 0.1 A g^−1^. In contrast, graphite demonstrated limited reversible capacity across all tested current densities, which fails to satisfy the requirements for advanced lithium-ion battery applications. For a clearer comparison of the rate performance, the first-cycle discharge capacities of the three materials across different rate conditions are presented in a bar chart (Figure 4f). The results clearly demonstrate that RC-Si/N delivers the highest reversible capacity, substantially outperforming both the etched RC-N sample and conventional graphite. The above test results reveal that the naturally occurring Si retained in the biomass carbon contributes significantly to the capacity.

Figure 4g compares the cycling stability over extended cycles of the three materials at a constant current density of 0.1 A g^−1^. The initial discharge/charge capacities are measured as 2357.7/1335.2 mAh g^−1^ for RC-Si/N, 1451.7/550.3 mAh g^−1^ for RC-N, and 195.8/160.0 mAh g^−1^ for graphite, respectively. The low initial CE of RC-Si/N and RC-N arises from their abundant surface area and numerous surface defects, which result in a large amount of lithium ions being irreversibly consumed during the formation of a thick SEI film in the initial cycle. After cycling for 200 cycles at 0.1 A g^−1^, RC-Si/N retains a high reversible capacity of 761.3 mAh g^−1^. For comparison, the RC-N electrode suffers from drastic capacity fading during the initial cycles. While graphite demonstrates stable cycling, its practical capacity remains considerably low.

To assess long-term capacity retention at high current densities, extended cycling performance (at 1, 2, and 5 A g^−1^) is displayed in Figure 4h–j. RC-Si/N delivers a capacity output of 374.9 mAh g^−1^ after 100 cycles at 1 A g^−1^, which increases to 517.7 mAh g^−1^ after 1000 cycles, indicating progressive electrode activation. By comparison, RC-N shows continuous capacity fading, retaining only 183.2 mAh g^−1^ after 1000 cycles. A similar trend is observed at 2 A g^−1^, where the capacity of RC-Si/N rises from 250.1 mAh g^−1^ after 100 cycles to 373.8 mAh g^−1^ after 1000 cycles, while RC-N declines to 158.9 mAh g^−1^ after 1000 cycles. Even at 5 A g^−1^, RC-Si/N exhibits a capacity of 171.1 mAh g^−1^ after 1000 cycles, outperforming RC-N (73.1 mAh g^−1^) under equivalent experimental conditions (Figure S2a,b). Figure 4k shows the discharge-specific capacity of the two anodes after cycling for 1000 cycles at various rates, along with the capacity of graphite after 100 cycles at the same rate conditions. It can be observed that the RC-Si/N anode still exhibits the highest capacity retention among the three materials during prolonged cycling under equivalent experimental settings. Moreover, among the three investigated materials, only RC-Si/N demonstrates a continuous increase in reversible capacity during extended cycling. This unique behavior can be attributed to the progressive activation of the carbon domains through repeated lithium-ion intercalation, the additional storage of lithium ions via the formation of defects and active sites during electrochemical cycling, and the adsorption of lithium ions not only on the carbon surface but also within the abundant micropores and mesopores of the hierarchical structure [58].

To evaluate the reliability of the data, three batches of batteries were tested, and for each batch, three individual batteries were examined under every condition. Figure S3a shows the discharge capacities of three batteries from the same batch when cycled at a current density of 0.1 A g^−1^ for 1, 100, and 200 cycles, respectively. Although the capacities of different batteries vary under the same test conditions, the batteries remain relatively stable, as supported by the relatively small standard deviation (Figure S3b). Figure S3c presents the test results from additional batches and more batteries. The detailed discharge capacity data and standard deviation results are shown in Table S3. It can be seen that the data for batteries from the same batch show relatively little fluctuation, while the differences between batteries from different batches are also not significant, with standard deviations ranging from 4.81 to 9.42. This thereby highlights the reliability of the results presented in this work.

To elucidate the electrode kinetics, EIS tests were conducted over a frequency range of 0.01 Hz to 100 kHz. The resulting Nyquist plots for the three materials are presented in Figure 4l, along with the corresponding equivalent circuit model. Each spectrum consists of a depressed semicircle in the high-to-medium frequency region, which reflects the charge transfer resistance (R_ct_) at the electrode-electrolyte interface, followed by a sloping line in the low-frequency region, representing solid-state ion diffusion behavior. The experimental data show excellent agreement with the fitted curves. In the equivalent circuit, R_s_ refers to the ohmic resistance associated with the electrolyte and electrical contacts. R_ct_ corresponds to the Faraday charge-transfer resistance, and W_o_ denotes the Warburg impedance associated with lithium-ion diffusion. Table S4 summarizes the corresponding fitting parameters, revealing a consistent resistance trend of RC-Si/N < RC-N < graphite. The significantly lower charge-transfer resistance of RC-Si/N indicates enhanced reaction kinetics, facilitating more efficient electron/ion transport across the electrode/electrolyte interface. The Li-ion diffusivity (D_Li_^+^) was further quantified to evaluate ionic transport behavior. Therefore, to obtain D_Li_^+^, it is necessary to analyze the low-frequency part of the curves using the following formula.

Z_re_ = R_s_ + R_ct_ + σω^−1/2^ (1)

D_Li_ = R^2^T^2^ (2A^2^n^2^F^4^C_Li_^2^σ^2^)^−1^ (2)

where ω and σ are the angular frequency and the Warburg coefficient, respectively; R, T, A, n, F, and C_Li_ represent the gas constant, absolute temperature, electrode area, number of electrons transferred, Faraday constant, and lithium-ion concentration in the electrolyte. The ω^−1/2^ versus Z’(Ω) plots obtained from the results in Figure S4 yielded slopes of 1366.5, 74.58, and 34.83, relating to σ values. Since D_Li_^+^ is inversely proportional to the square of σ, RC-Si/N exhibits the highest Li-ion diffusivity among the three materials [59,60]. These collective findings confirm that RC-Si/N possesses superior lithium-ion diffusivity, reversible capacity, and robust cycling endurance, outperforming both its RC-N and conventional graphite.

**Figure 5:**
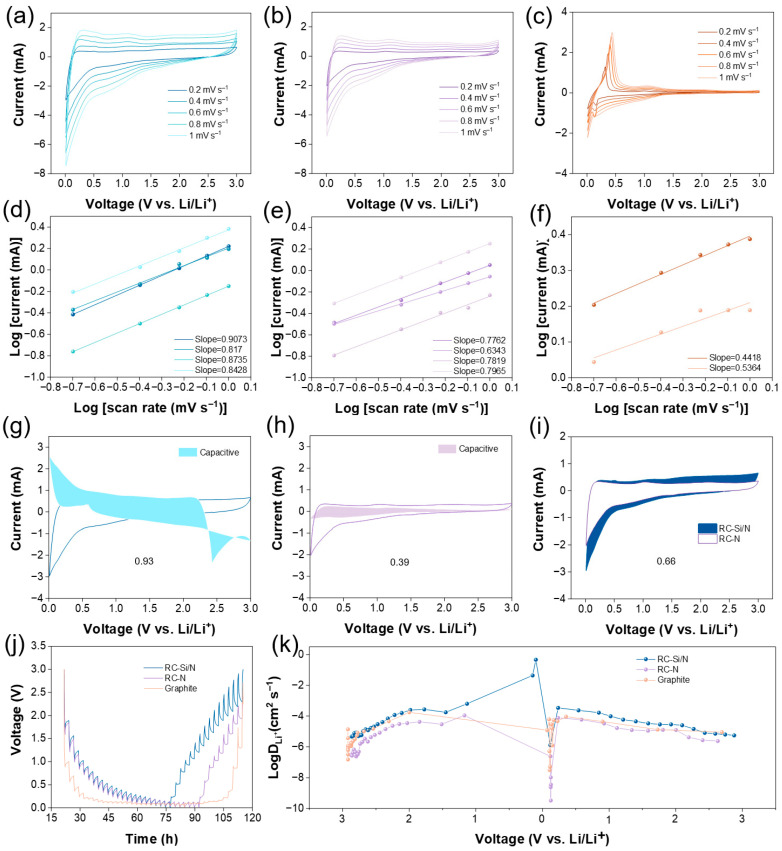
Dynamic characterization: The CV curves of (**a**) RC-Si/N, (**b**) RC-N and (**c**) Graphite anodes at 0.2^−1^ mV s^−1^; The linear fitting of log(i) versus log(v) for the cathodic and anodic peaks of (**d**) RC-Si/N, (**e**) RC-N, and (**f**) Graphite; Typical pseudocapacitance determination of (**g**) RC-Si/N, (**h**) RC-N at 0.2 mV s^−1^; (**i**) CV curves of RC-Si/N and RC-N at 0.2 mV s^−1^; (**j**) GITT profiles and (**k**) calculated Li^+^ diffusion coefficients of experimental anodes.

Figure 5a–c present the CV profiles of RC-Si/N, RC-N, and graphite at 0.2–2 mV s^−1^ over a voltage window of 0.01–3.00 V, characterizing their respective lithium storage behaviors. For RC-Si/N (Figure 5a), the anodic peak observed at approximately 0.5 V corresponds to the dealloying process of Li_x_Si, forming amorphous silicon. The consistent position of this peak across increasing scan rates indicates the stable activation of silicon during cycling [61]. This characteristic peak is absent in the RC-N sample in Figure 5b, which can be attributed to its lower silicon content, making it insufficient to drive the alloying/dealloying reaction. Additional redox features around 0.6, 1.0, 1.5, and 2.0 V are associated with electrode/electrolyte interfacial parasitic reactions and the delithiation of lithium-carbon composites [62]. In contrast, the graphite electrode exhibits typical redox pairs near 0.2 V and 0.4 V, corresponding to the staged lithium intercalation and deintercalation in the graphite layers. To further quantify the electrochemical kinetics, the current response (i) was analyzed using the power-law relationships expressed in Equations (3) and (4).

i = av^b^ (3)

log(i) = b log(v) + log(a)(4)

where a and b are adjustable parameters, and v is the scanning rate. The b values derived from the gradient of the log(i) versus log(v) plots, transformed from the above equations, provide insight into the charge storage behavior. A b value near 0.5 relates to diffusion-governed intercalation, while a b value approaching 1.0 indicates surface-controlled capacitive behavior. As displayed in Figure 5d, the characteristic redox peaks of RC-Si/N are associated with b-values of 0.9073, 0.817, 0.8735, and 0.8428, confirming that its capacity is predominantly governed by pseudocapacitive processes. In comparison, RC-N exhibits b-values of 0.7762, 0.6343, 0.7819, and 0.7965 in Figure 5e, reflecting a mixed mechanism with both capacitive and diffusion-controlled contributions. By contrast, graphite shows b-values of 0.4418 and 0.5364 in Figure 5f, consistent with its characteristic diffusion-dominated lithium intercalation mechanism. These results quantitatively demonstrate that the Si/N co-doping in RC-Si/N effectively enhances surface-driven charge storage, leading to superior rate performance. The capacitive contributions of RC-Si/N and RC-N were further quantified using Equation (5) [63,64].

i(v) = k_1_v + k_2_v^1/2^ (5)

As illustrated in Figure 5g,h, the pseudocapacitive contribution percentage at 0.2 mV s^−1^ reaches 93% for RC-Si/N, significantly higher than the 39% observed for RC-N. This dominant surface-controlled charge storage mechanism explains the excellent rate capability and cycling stability of RC-Si/N under high-current conditions, which aligns with the performance trends in Figure 4. A comparison of the CV curve areas at 0.2 mV s^−1^ provides additional evidence for the enhanced capacity of RC-Si/N in Figure 5i. When normalized with the area of RC-Si/N set as 1, the integrated area for RC-N is only 0.66, indicating a substantially higher lithium storage capacity for the Si/N co-doped material. GITT measurements were performed to evaluate lithium-ion diffusion kinetics during discharge/charge processes at 100 mA g^−1^, using 10 min current pulses separated by 30 min rest periods, as shown in Figure 5j. The corresponding lithium-ion diffusion coefficients, derived from the GITT data and presented as a function of voltage in Figure 5k, confirm that RC-Si/N exhibits the highest Li^+^ migration rate among the three materials. This result is fully consistent with the electrochemical performance data and further validates the kinetic advantages of the RC-Si/N electrode.

Table S2 summarizes the Li storage properties of biomass-derived carbon-based anode materials by different synthesis routes in recent years [24,47,48,49,50,51,52,53,54]. As can be seen from the table, in terms of process complexity, cost, environmental impact, and final lithium storage performance, the routes presented in this paper all demonstrate excellent levels, indicating their potential for promising applications. The good lithium storage performance of the natural Si/N dual-element co-doped biomass-derived carbon micron-tubes can be ascribed to the following factors. Firstly, the incorporation of N elements into the carbon lattice introduces additional electron donors, which improves the overall electrical conductivity, facilitating faster electron transport during charge/discharge cycles. Secondly, both silicon and nitrogen doping create numerous defects and active sites within the carbon structure, providing more sites for lithium-ion adsorption and insertion, thereby significantly enhancing the specific capacity. Thirdly, co-doping of Si and N can induce a unique electronic structure and surface chemistry, potentially enhancing the binding energy with lithium ions and promoting more efficient and reversible lithium storage compared to single-element doping. Fourthly, the one-dimensional hollow tubular architecture offers a short diffusion pathway for lithium ions and electrons, while also providing a high electrochemically active area that maximizes the electrode/electrolyte contact, leading to improved rate capability. Finally, the robust carbon matrix derived from biomass, combined with the micron-tube structure, effectively accommodates the mechanical stress and volume changes associated with lithium insertion/extraction, ensuring superior cycling stability and long-term durability.

### 3.3. DFT Calculations

**Figure 6:**
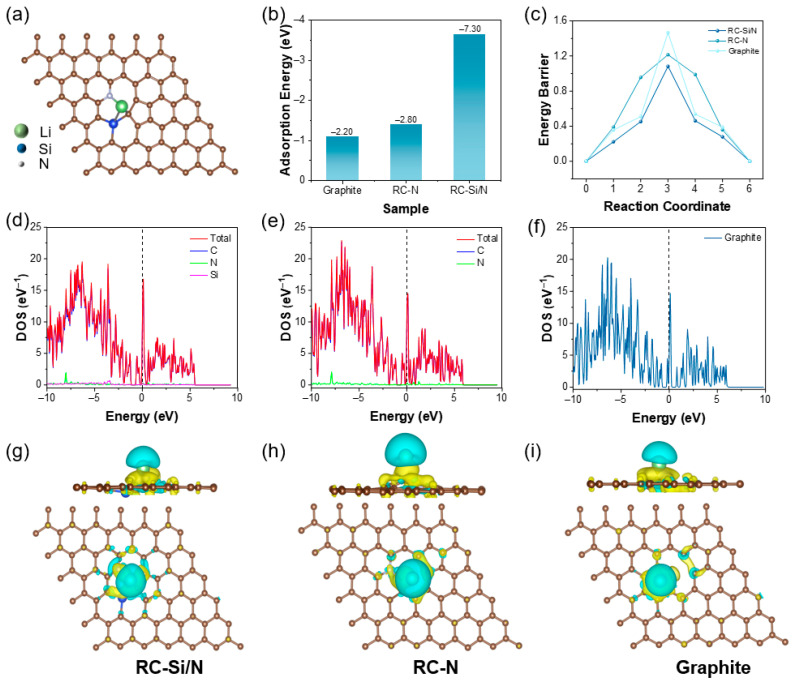
DFT theoretical calculation: (**a**) DFT calculation model of RC-Si/N; (**b**) The calculation towards the adsorption energy of Li on the surface of RC-Si/N, RC-N and Graphite; (**c**) The variations in migration energy during the diffusion process of RC-Si/N, RC-N and Graphite; (**d**–**f**) Project DOS of RC-Si/N, RC-N and Graphite; Differential charge of (**g**) RC-Si/N, (**h**) RC-N and (**i**) Graphite.

To gain deeper insight into the influence of silicon and nitrogen doping on lithium storage behavior, density functional theory (DFT) calculations were conducted on the three electrode materials. Figure 6a displays the optimized atomic model. The modeling is based on the identification of C–N and Si–C chemical bonds from XPS results, as well as the confirmation of disordered carbon and defects from Raman spectroscopy and TEM images. Accordingly, on the basis of carbon material modeling, the above conditions were introduced, followed by model optimization, ultimately yielding the RC-Si/N model after Li atom adsorption (Figure 6a). The differential charge density diagrams in Figure 6g–i reveal charge redistribution upon lithium adsorption, with yellow and cyan regions representing localized charge enrichment and depletion. The pronounced charge transfer toward the lithium atom demonstrates a strong adsorption interaction, which is most evident in RC-Si/N, followed by RC-N and graphite. This trend is quantitatively corroborated by the calculated lithium adsorption energies in Figure 6b: −7.3 eV for RC-Si/N, −2.8 eV for RC-N, and −2.2 eV for graphite, confirming the superior lithiophilicity of the co-doped material [65,66]. The PDOS plots in Figure 6d–f further elucidate the electronic structure modifications. The Si/N co-doped system exhibits a markedly higher density of states near the Fermi level compared with RC-N and graphite, indicating enhanced electronic conductivity and more favorable lithium-ion adsorption and transport kinetics. Furthermore, as depicted in the lithium-ion migration energy profile (Figure 6c), RC-Si/N shows consistently lower diffusion energy barriers across all intermediate states, suggesting facilitated Li^+^ transport within its structure. Collectively, these computational results provide a theoretical foundation for the exceptional electrochemical performance of the RC-Si/N anode [67,68,69].

### 3.4. Post-Cycling Characterization

**Figure 7:**
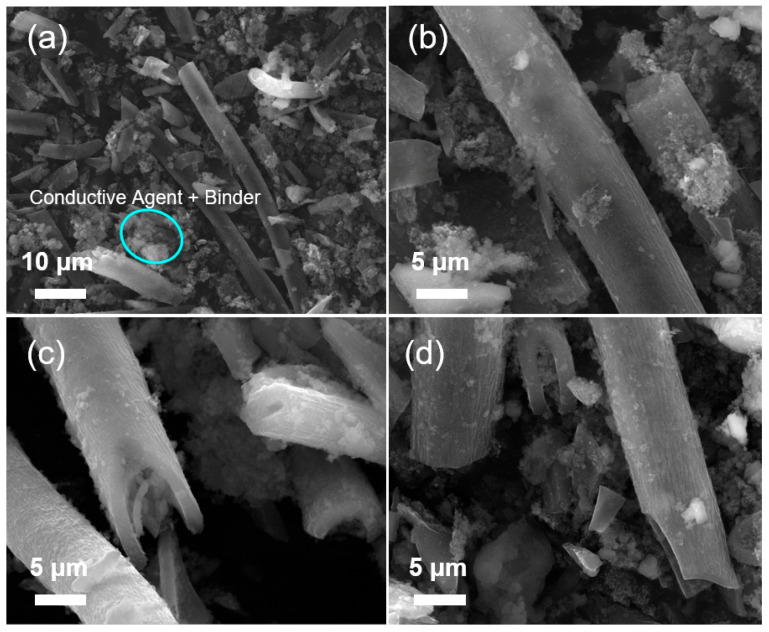
SEM images of RC-Si/N after cycling for (**a**,**b**) 100 cycles, (**c**) 500 cycles, and (**d**) 1000 cycles at 1 A g^−1^.

Post-cycling characterization was undertaken to examine the structural and chemical robustness of the RC-Si/N material after cycling at 1 A g^−1^ for different cycles. Figure 7 shows SEM images of the RC-Si/N electrodes after cycling for 100 (Figure 7a,b), 500 (Figure 7c), and 1000 (Figure 7d) cycles. It can be seen that the carbon-based micron-tubes are mixed with the conductive agent and binder on the electrode plate. After multiple cycles, the morphology of the micron-tubes presents good stability (without any obvious fractures or collapses) compared with the pristine material, demonstrating the excellent mechanical toughness of the material during the repeated Li^+^ insertion/extraction process.

**Figure 8:**
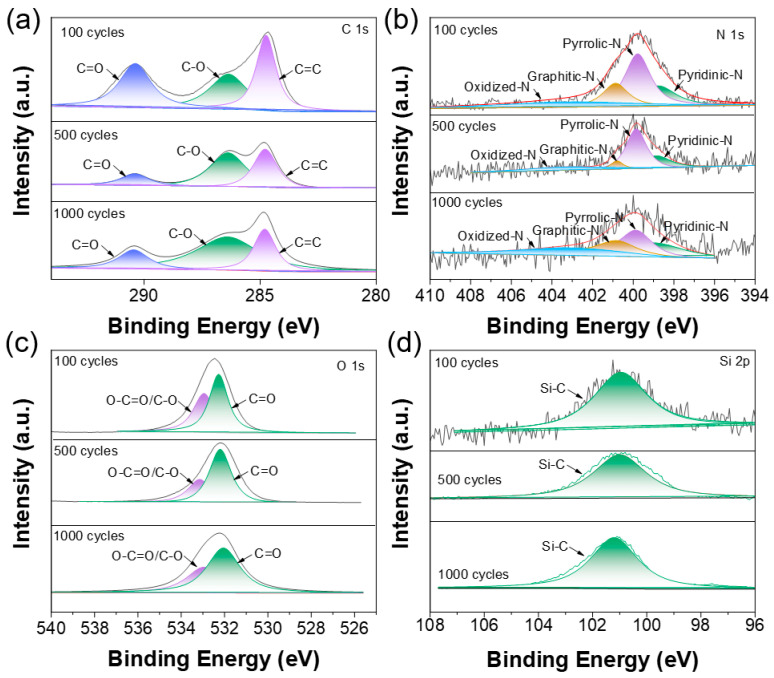
XPS spectra of RC-Si/N after cycling for 100 cycles, 500 cycles and 1000 cycles at 1 A g^−1^: (**a**) C 1s, (**b**) N 1s, (**c**) O 1s and (**d**) Si 2p.

XPS analysis of the cycled electrode in Figure 8 reveals an evolution in surface chemistry. Compared with the pristine material, the electrode subjected to 100 cycles shows certain changes in the relative intensities of the XPS peaks. The C 1s spectrum (Figure 8a) demonstrates a noticeable redistribution in the relative intensities of the C=C, C-O, and C=O components, suggesting structural rearrangement resulting from Li^+^ insertion and removal. The chemical states of nitrogen (Figure 8b) and silicon (Figure 8d) remain largely unchanged, indicating the robust stability of the Si/N-C coordination environment. However, the O 1s spectrum (Figure 8c) exhibits a significant decrease in the -COOH contribution, accompanied by a relative increase in the C=O and C-O/O-C=O species. This transformation originates from the electrochemical oxidation of carboxyl groups at higher operating potentials, leading to their conversion into more stable oxygen-containing functional groups through side reactions with the electrolyte. In addition, it can be observed that as the number of cycles increases to 500 and then to 1000, the intensity of the peaks slightly decreases. Specifically, the binding energy of Si 2p (Figure 8d) shows a tendency to shift toward higher binding energy, indicating that the structure of the material is at risk of degradation during long-term cycles. However, under the current conditions, the relevant structures can still maintain a relatively stable state. These findings collectively underscore the interfacial stability and structural retention of RC-Si/N, consistent with its outstanding electrochemical durability [70].

## 4. Conclusions

Si/N co-doped carbon micron-tubes were successfully synthesized from biomass reed catkins precursors via a combined chemical activation and two-step calcination method. The resulting architecture possesses a high specific surface area and well-developed porosity, offering substantial active sites for Li-ion storage. At the same time, the hollow tubular structure facilitates efficient ion transport. The synergistic presence of trace Si and N significantly enhances the reversible capacity, contributing to relatively good electrochemical performance. Serving as a LIB anode, RC-Si/N delivers a good recoverable capacity of 761.3 mAh g^−1^ after 200 cycles at 100 mA g^−1^, and maintains remarkable cyclability with discharge capacities of 517.7 mAh g^−1^ after 1000 cycles at 1 A g^−1^ and 373.8 mAh g^−1^ at 2 A g^−1^, surpassing most recently reported biomass-derived carbons. DFT calculations corroborate that RC-Si/N possesses the strongest Li adsorption energy (−7.3 eV) and the lowest Li^+^ migration barrier among the studied systems, providing a theoretical basis for its exceptional reversible capacity and fast reaction kinetics. These findings collectively affirm that RC-Si/N is a highly attractive and low-cost anode candidate for advanced LIBs.

## Data Availability

The original contributions presented in this study are included in the article/Supplementary Material. Further inquiries can be directed to the corresponding author.

## Supplementary Materials

The following supporting information can be downloaded at https://www.mdpi.com/article/10.3390/ma19142951/s1: Figure S1: SEM image (a) and (b–f) EDS mapping of RC-N. Figure S2: Long-term cycling performance of (a) RC-N and (b) Graphite anode at 5 A g^−1^. Figure S3: Electrochemical stability tests of the batteries. Figure S4: The relationship between Z_re_ and ω^−1/2^ in the low-frequency region of RC-Si/N, RC-N and Graphite. Table S1: Silicon contents in RC-Si/N and RC-N. Table S2: Li storage properties of biomass carbon materials synthesized by different routes. Table S3: Electrochemical stability of three batches of batteries. Table S4: Simulated data of the EIS results.
