# Supplementary material for: Natural Si/N Co-Doped Porous Biomass Carbon Micron-Tubes as High-Performance Anode Materials for Lithium-Ion Batteries
Source: Materials (Basel). 2026 Jul 9;19(14):2951. doi: 10.3390/ma19142951 (PMC13412887; doi:10.3390/ma19142951)
Supplement: Supplementary file 1 [file materials-19-02951-s001.zip › materials-4382072-supplementary.pdf]

Supporting information

Natural Si/N co-Doped Porous Biomass Carbon Micron-Tubes as High-Performance Anode Materials for Lithium-Ion Batteries

Ziqing Xu, Kai Cao, and Zhifeng Wang *

"The Belt and Road Initiative" Advanced Materials International Joint Research Center of Hebei Province, School of Materials Science and Engineering, Hebei University of Technology, Tianjin 300401, China;

* Correspondence: wangzf@hebut.edu.cn (Z.W.); Tel.: +86-22-6020-2006 (Z.W.)

S1 Experimental Details

Material Characterization. The crystalline structures of the synthesized samples were characterized by X-ray diffraction (XRD) using a Smart Lab diffractometer (Rigaku, Tokyo, Japan) with Cu Kα radiation. Morphological and microstructural analyses were performed using a scanning electron microscope (SEM, Quanta 450 FEG, FEI, Hillsboro, OR, USA) and a transmission electron microscope (TEM, JEM-2010, JEOL, Tokyo, Japan). Chemical composition and elemental valence states were determined by X-ray photoelectron spectroscopy (XPS, Waltham, MA, USA) on a Thermo Scientific K-Alpha spectrometer. Specific surface area and pore size distribution were evaluated through nitrogen adsorption-desorption measurements at 77 K using a Micromeritics ASAP 2460 analyzer (Micromeritics, Norcross, GA, USA). Raman spectra were acquired in the range of 100–2000 cm^{-1} with a Horiba Scientific spectrometer (HORIBA, Tokyo, Japan). Elemental quantification was carried out by inductively coupled plasma optical emission spectrometry/mass spectrometry (ICP-OES/MS) using an Agilent 7800 system (San Jose, CA, United States).

Electrochemical Measurements. Electrode plates were fabricated by coating a homogeneous slurry onto a copper foil current collector. The slurry consisted of active material, polyvinylidene fluoride binder, and Super P conductive carbon in a mass ratio of 8:1:1, dissolved in N-methyl-2-pyrrolidone. The coated foils were subsequently vacuum-dried at 60 °C for 12 hours. CR2032 coin-type half-cells were then assembled in an argon-filled glove box, employing Celgard 2325 as the separator and 1.0 M $LiPF_6$ in a mixture of ethylene carbonate and diethyl carbonate as the electrolyte. The electrochemical performance of the RC-Si/N, RC-N, and graphite anodes was evaluated using a NEWARE CT-4000 battery testing system (Shenzhen, China). All galvanostatic cycling tests were conducted within a voltage window of 0.01–3.0 V (vs. Li^+/Li). Electrochemical impedance spectroscopy (EIS) measurements were also performed to characterize the interfacial charge-transfer resistance (CH660E, Shanghai, China).

Calculation Method. All density functional theory (DFT) calculations were performed using the Projected Augmented Wave (PAW) method as implemented in the DS-PAW software package (HZWTECH, version 2022A, Shanghai, China). The exchange-correlation interactions were treated using the Perdew-Burke-Ernzerhof (PBE) functional within the generalized gradient approximation (GGA), augmented with the Grimme's D3 (DFT-D3) dispersion correction to account for van der Waals interactions. To balance computational accuracy and efficiency, we established a supercell model in the simulation that contained 90 carbon atoms, 1 silicon atom, and 1 nitrogen atom (including multiple carbon vacancies to simulate the disorder in real biomass carbon, and only the most critical active area is shown in the image). The Broyden-Fletcher-Goldfarb-Shanno (BFGS) algorithm is employed for geometric structure optimization. The model size is 6 × 6 carbon supercells (C 002) which are designed to accommodate the doping sites for Si and N, and to ensure that the Li atoms in adjacent supercells do not interfere with each other. The truncation energy is 500 eV, which is highly convergent for the plane wave basis sets of C, N, Si, and Li elements, ensuring the accuracy of the total energy calculation. Using a 5 × 5 × 1 Monkhorst-Pack grid for sampling the Brillouin zone of a 6 × 6 unit cell is already very dense, which is sufficient to obtain smooth charge density and energy. Additionally, the 15 Å vacuum layer ensures weak interactions between the periodic mirrors in the Z direction.

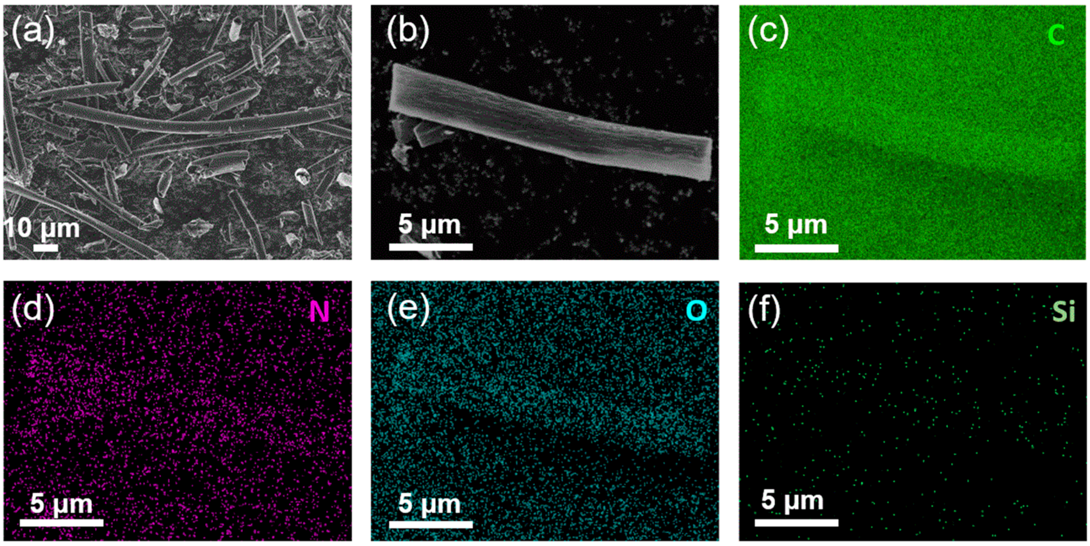

Figure S1. SEM image (a) and (b-f) EDS mapping of RC-N.

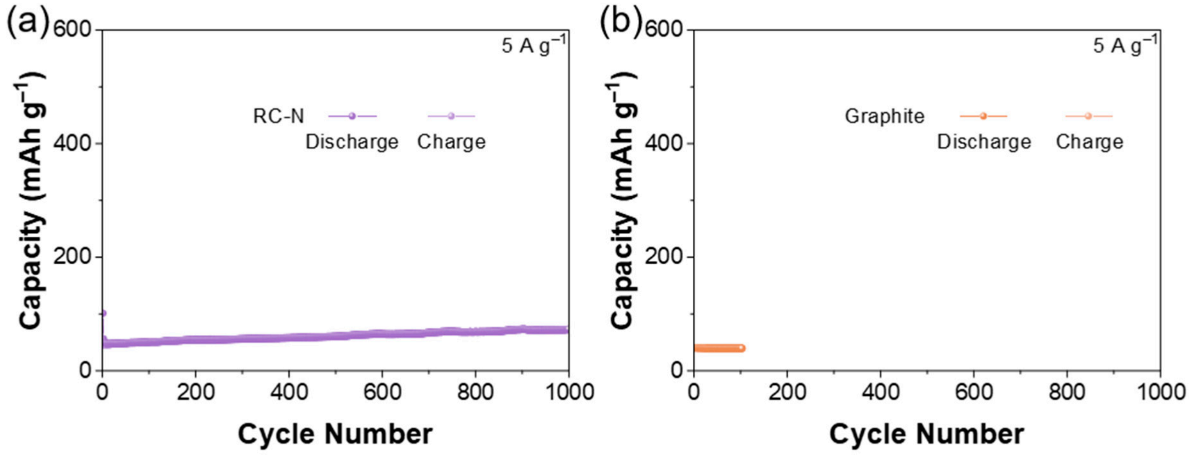

Figure S2. Long-term cycling performance of (a) RC-N and (b) Graphite anode at 5 A g^{-1}.

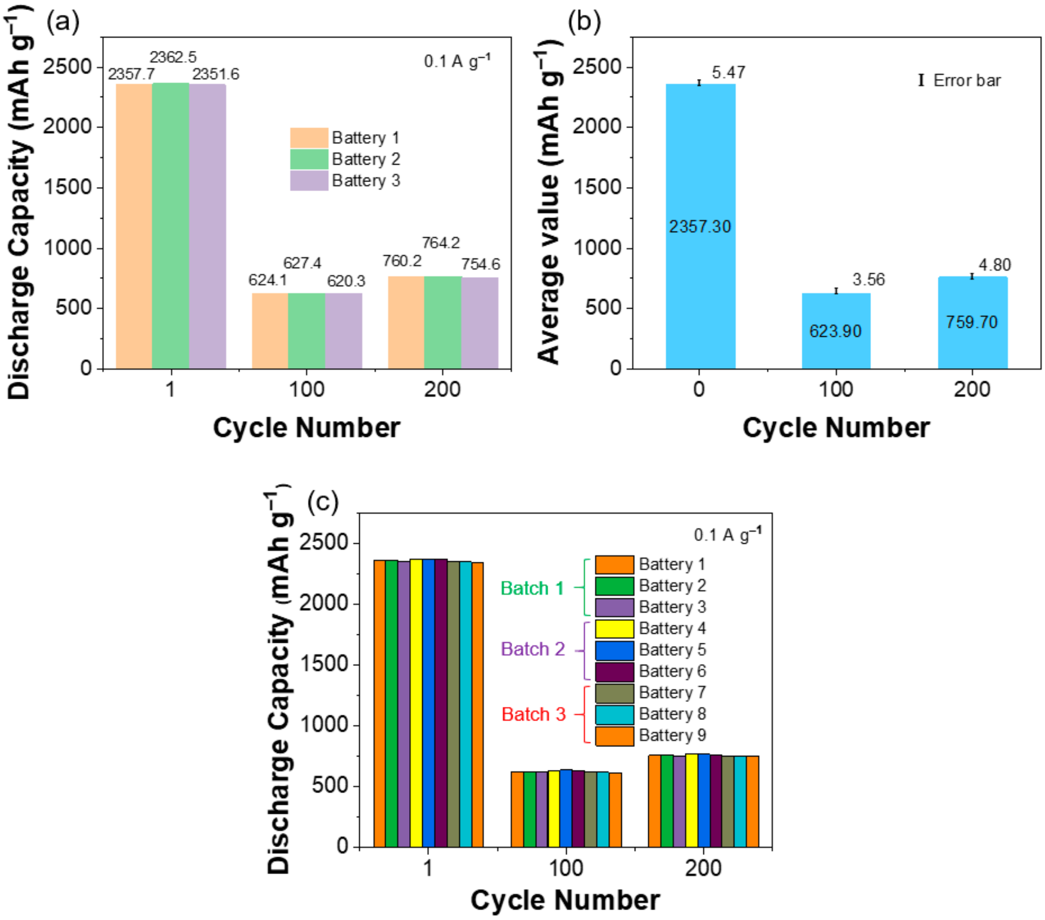

Figure S3. Electrochemical stability tests of the batteries. (a) Discharge specific capacities of the three batteries from the same batch at 0.1 A g^{-1}; (b) Standard deviations of the data in (a) at different cycle numbers; (c) Electrochemical stability of three groups of batteries from different batches.

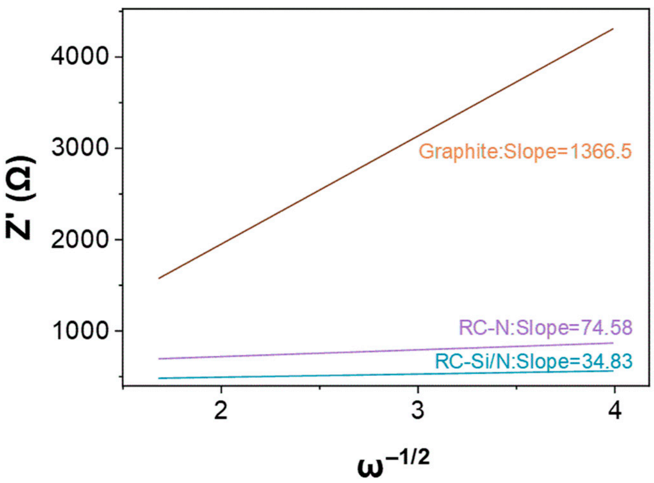

Figure S4. The relationship between Z_{re} and $\omega^{-1/2}$ in the low-frequency region of RC-Si/N, RC-N and Graphite.

Table S1 Silicon contents in RC-Si/N and RC-N.

Sample	W (%)			Standard deviations
	Batch 1	Batch 2	Batch 3	
RC-Si/N	1.878	1.882	1.873	0.0045
RC-N	0.019	0.019	0.018	0.0006

Table S2. Li storage properties of biomass carbon materials synthesized by different routes.

Anode materials	Synthesis method	Process Complexity	Cost	Environmental Impact	ICE (%)	Current density (mA g⁻¹)	Cycle number	Reversible capacity (mAh g⁻¹)	Ref
Micron-sized carbon particles	Hydrothermal method and high-temperature carbonization	**Moderate** (Two-step process, standard equipment)	**Moderate** (energy-intensive carbonization; hydrothermal reactor required)	**Moderate** (hydrothermal waste liquid needs treatment; CO_2 emissions from high-temperature calcination)	45.5	100<br>500	1<br>500	428<br>234	[47]
Mesoporous carbon	Template method and activation method	**High** (Multiple steps; template removal is tedious and time-consuming)	**High** (expensive templates; large consumption of acids/bases for template removal)	**High** (strong acid/base wastewater; high reagent consumption; significant chemical waste)	46.6	100<br>1000	500<br>500	365<br>112.6	[48]
N-doped porous carbon	Carbonization and KOH activation	**Moderate** (conventional two-step process; well-established technique)	**Moderate** (KOH is relatively cheap but consumed in large amounts; high energy for carbonization)	**Moderate** (KOH wastewater requires neutralization; large water consumption for washing)	50.9	100<br>1000	650<br>1000	393<br>155	[49]
Hierarchical N/O co-doped carbon	Calcining and pickling	**Low** (simple process; minimal steps; no complex equipment)	**Low** (few reagents; low energy consumption; cost-effective)	**Moderate** (acidic pickling waste requires neutralization; calcination generates exhaust gases, but overall reagent usage is small)	-	200<br>1000	1<br>500	311.8<br>307.4	[50]
Porous carbon spheres	Hydrothermal and chemical activation	**Moderate** (hydrothermal + activation; requires high-pressure autoclave)	**Moderate** (autoclave equipment; chemical activators add to material cost)	**Moderate** (hydrothermal and activation waste liquids; moderate energy consumption)	-	500<br>1000	10<br>10	307<br>245	[51]
N-doped carbon nanofiber	Purification and carbonization	**High** (nanofiber fabrication requires special equipment and precise process control)	**High** (specialized setup; purification solvents add cost; energy-intensive)	**Low to Moderate** (limited wastewater; mainly gaseous emissions from carbonization; solvent recovery possible)	45.7	1000<br>1500	500<br>5	174<br>215.6	[52]
Carbon@SiO_2 nanotubes	Coating and carbonization	**High** (nanotube synthesis is complex; coating uniformity is difficult to control)	**High** (organic precursors and coating materials are expensive; multi-step operation)	**Moderate** (organic solvents used in coating; carbonization generates VOC emissions; manageable with proper treatment)	-	100<br>1000	300<br>800	815<br>353	[53]
Porous carbon@ $ZnMn_2O_4$	Carbonization, chemical activation and complexation	**Very High** (three-step process: carbonization + activation + complexation; most complicated)	**Very High** (multiple reagents; Zn/Mn precursors are costly; longest processing time)	**Very High** (heavy metals Zn and Mn pose serious environmental risks; wastewater requires stringent treatment)	-	100<br>1000	200<br>1500	197.9<br>244.7	[54]
Hierarchical porous structure carbon	Carbonization and KOH activation	**Moderate** (conventional two-step process; well-established technique)	**Moderate** (KOH is relatively cheap but consumed in large amounts; high energy for carbonization)	**Moderate** (KOH wastewater requires neutralization; large water consumption for washing)	-	100<br>5000	100<br>10	700<br>146	[55]

RC-Si/N	Carbonization and KOH activation	**Moderate** (conventional two-step process; well-established technique)	**Moderate** (KOH is relatively cheap but consumed in large amounts; high energy for carbonization)	**Moderate** (KOH wastewater requires neutralization; large water consumption for washing)	56.6	100<br>1000	200<br>1000	761.3<br>517.7	This work

Table S3. Electrochemical stability of three batches of batteries.

Current density ($A g^{-1}$)	Cycle number	Batch 1					Batch 2					Batch 3					All batches	
		Battery 1	Battery 2	Battery 3	AV	SD	Battery 4	Battery 5	Battery 6	AV	SD	Battery 7	Battery 8	Battery 9	AV	SD	AV	SD
0.1	1	2357.7	2362.5	2351.6	2357.3	5.47	2369.4	2373.3	2365.5	2369.4	3.9	2355.5	2349.8	2342.3	2349.2	6.6	2358.6	9.42
	100	624.1	627.4	620.3	623.9	3.56	635.6	640.7	634.3	636.9	3.37	619.7	626.5	614.4	620.2	6.07	627	8.32
	200	760.2	764.2	754.6	759.7	4.8	769.4	770.3	763.2	767.6	3.92	753.2	757.4	749.8	753.5	3.81	760.3	6.77
1	1	1939.2	1945.3	1936.9	1940.5	4.37	1935.2	1942.6	1947.6	1941.8	6.21	1940.2	1933.7	1932.6	1935.5	4.24	1939.3	5.13
	500	480.8	486.3	477.2	481.4	4.6	478.9	485.6	487.1	483.9	4.4	482.4	476.3	471.9	476.9	5.27	480.7	4.83
	1000	517.7	521.3	513.8	517.6	3.77	520.6	530.2	524.8	525.2	4.82	515.1	512.3	509.8	512.4	2.65	518.4	6.12
2	1	1932.3	1941.3	1930.1	1934.6	6.19	1934.9	1946.4	1930.2	1937.2	8.38	1931.2	1928.7	1926.6	1928.8	2.3	1933.5	6.4
	500	267.9	270.6	263.4	267.3	3.64	272.3	279.4	270.1	273.9	4.93	266.5	263.9	259.8	263.4	3.35	268.2	5.78
	1000	373.8	381.3	375.7	376.9	3.9	381.5	384.6	376.3	380.8	4.2	376.2	372.7	368.4	372.4	3.91	376.7	4.81
Note:	AV: average value; SD: standard deviation																	

Table S4. Simulated data of the EIS results.

Sample	$R_s(\Omega)$	$R_{ct}(\Omega)$
RC-Si/N	3.5	345.4
RC-N	2.2	477.9
Graphite	3.5	709.3
